# *Aspergillus* Cell Wall Chitin Induces Anti- and Proinflammatory Cytokines in Human PBMCs via the Fc-γ Receptor/Syk/PI3K Pathway

**DOI:** 10.1128/mBio.01823-15

**Published:** 2016-05-31

**Authors:** K. L. Becker, V. Aimanianda, X. Wang, M. S. Gresnigt, A. Ammerdorffer, C. W. Jacobs, R. P. Gazendam, L. A. B. Joosten, M. G. Netea, J. P. Latgé, F. L. van de Veerdonk

**Affiliations:** a Department of Internal Medicine and Radboud Center for Infectious Diseases (RCI), Radboud University Medical Center, Nijmegen, The Netherlands; b Unité des Aspergillus, Institut Pasteur, Paris, France; c Sanquin Research and Landsteiner Laboratory, Academic Medical Center, University of Amsterdam, Amsterdam, The Netherlands

## Abstract

Chitin is an important cell wall component of *Aspergillus fumigatus* conidia, of which hundreds are inhaled on a daily basis. Previous studies have shown that chitin has both anti- and proinflammatory properties; however the exact mechanisms determining the inflammatory signature of chitin are poorly understood, especially in human immune cells. Human peripheral blood mononuclear cells were isolated from healthy volunteers and stimulated with chitin from *Aspergillus fumigatus*. Transcription and production of the proinflammatory cytokine interleukin-1β (IL-1β) and the anti-inflammatory cytokine IL-1 receptor antagonist (IL-1Ra) were measured from the cell culture supernatant by quantitative PCR (qPCR) or enzyme-linked immunosorbent assay (ELISA), respectively. Chitin induced an anti-inflammatory signature characterized by the production of IL-1Ra in the presence of human serum, which was abrogated in immunoglobulin-depleted serum. Fc-γ-receptor-dependent recognition and phagocytosis of IgG-opsonized chitin was identified as a novel IL-1Ra-inducing mechanism by chitin. IL-1Ra production induced by chitin was dependent on Syk kinase and phosphatidylinositol 3-kinase (PI3K) activation. In contrast, costimulation of chitin with the pattern recognition receptor (PRR) ligands lipopolysaccharide, Pam3Cys, or muramyl dipeptide, but not β-glucan, had synergistic effects on the induction of proinflammatory cytokines by human peripheral blood mononuclear cells (PBMCs). In conclusion, chitin can have both pro- and anti-inflammatory properties, depending on the presence of pathogen-associated molecular patterns and immunoglobulins, thus explaining the various inflammatory signatures reported for chitin.

## IMPORTANCE

Invasive aspergillosis and allergic aspergillosis are increasing health care problems. Patients get infected by inhalation of the airborne spores of *Aspergillus fumigatus*. A profound knowledge of how *Aspergillus* and its cell wall components are recognized by the host cell and which type of immune response it induces is necessary to develop target-specific treatment options with less severe side effects than the treatment options to date. There is controversy in the literature about the receptor for chitin in human cells. We identified the Fc-γ receptor and Syk/PI3K pathway via which chitin can induce anti-inflammatory immune responses by inducing IL-1 receptor antagonist in the presence of human immunoglobulins but also proinflammatory responses in the presence of bacterial components. This explains why *Aspergillus* does not induce strong inflammation just by inhalation and rather fulfills an immune-dampening function. While in a lung coinfected with bacteria, *Aspergillus* augments immune responses by shifting toward a proinflammatory reaction.

## INTRODUCTION

Conidia of the opportunistic fungus *Aspergillus fumigatus* are responsible for allergic syndromes, especially allergic bronchopulmonary aspergillosis. This occurs especially in patients with asthma or cystic fibrosis, who are chronically colonized with *Aspergillus* (1). Several *Aspergillus* proteins are known as allergens, driving pathology in allergic bronchopulmonary aspergillosis. In addition to proteins, the polysaccharide chitin is also known to induce allergy by causing accumulation of interleukin-4 (IL-4)-expressing innate immune cells (2). Humans do not produce chitin but express proteins that can degrade or bind chitin, such as chitinases and chitinase-like proteins, respectively (3). Polymorphisms in chitinase genes (4) or elevated serum levels of chitinase-like proteins have been associated with asthma (5), although the exact mechanism describing how chitinases and chitin interact with immune cells and trigger immune responses is not yet completely understood.

Chitin is one of the major fibrillar components of the *Aspergillus* cell wall and is covalently bound to β-(1,3)-glucans (6). While in a resting conidium, polysaccharides are shielded by a rodlet and melanin layer; during germination chitin is exposed on the mycelium surface and can interact with cells of the innate immune system (6). *Aspergillus* cell wall polysaccharides serve as pathogen-associated molecular patterns (PAMPs), which are recognized by pattern recognition receptors (PRRs) to induce an innate immune response with consequent induction and shaping of an adaptive immune response (7). While β-(1,3)-glucan is known as the ligand for dectin-1 (8), recent studies propose different identities of the chitin recognition receptor and the induced immune response. Schlosser et al. identified FIBCD1 as a high-affinity chitin binding receptor of the intestine that controls immune responses against ingested parasites and fungi (9). Additionally, chitin was described to have proinflammatory properties by inducing IL-17 via the Toll-like receptor 2 (TLR2) pathway (10) in murine cells, and a recent study suggested recognition of chitin via the mannose receptor (MR) on the surface of the phagocytes and by the nucleotide-binding oligomerization domain 2 (NOD2) and TLR9 receptors in the cytoplasm, leading to the induction of the anti-inflammatory cytokine IL-10 in mouse macrophages (11). No pattern recognition receptor or signaling pathway triggered by chitin has been identified in human immune cells to date. In the present study, we aimed to elucidate the immunological properties of chitin isolated from the *A. fumigatus* cell wall and its receptor in human peripheral blood mononuclear cells (PBMCs) and the mechanism and circumstances under which chitin can switch between anti-inflammatory immune responses toward proinflammatory responses.

## RESULTS

### *Aspergillus fumigatus* cell wall chitin produces IL-1Ra in the presence of serum.

**FIG 1:**
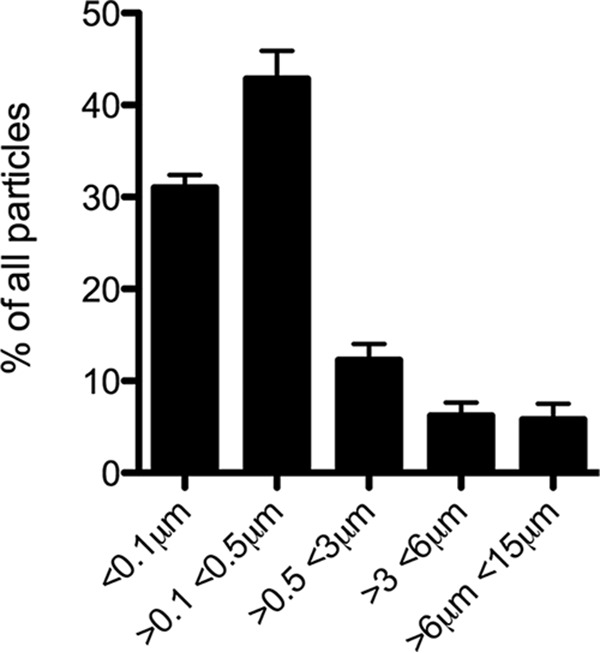
Characterization of chitin particles purified from *Aspergillus* mycelium. Chitin particles were measured by flow cytometry and compared with reference beads; the distribution of chitin particles was calculated. Means and standard deviations from 5 independent measurements of three different batches of *Aspergillus* chitin are depicted.

**FIG 2:**
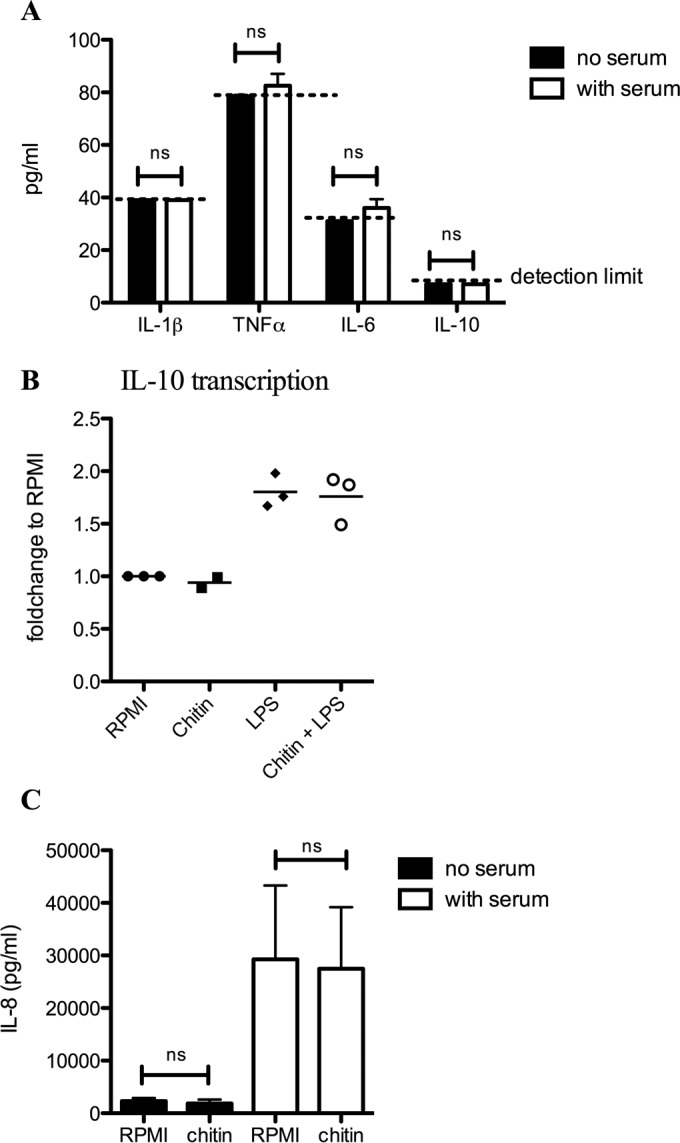
*Aspergillus* chitin does not induce proinflammatory cytokines or the anti-inflammatory cytokine IL-10. (A) IL-1β, TNF-α, IL-6, and IL-10 production (*n* = 6), (C) IL-8 production (*n* = 8), and (B) IL-10 transcription (*n* = 3), were measured either in the cell culture supernatant (A and C) or in mRNA (B) of PBMCs of healthy controls that were stimulated (A and C) with *Aspergillus* chitin in the presence of either medium (black bars) or human serum (white bars) or (B) with *Aspergillus* chitin or LPS alone or LPS plus *Aspergillus* chitin. ns, not significant.

**FIG 3:**
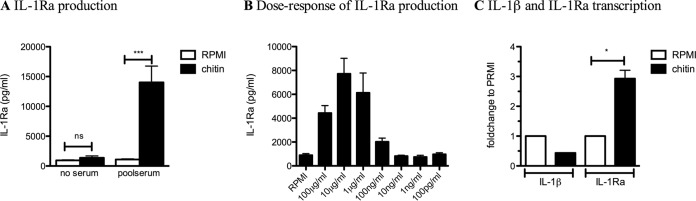
Chitin induces IL-1Ra release and transcription of IL-1Ra but not IL-1β in the presence of human serum. (A and B) IL-1Ra production measured in the cell culture supernatant of PBMCs of healthy controls after 24 h of stimulation in the presence of either RPMI (white bars) or human serum (black bars) (A) with 10 µg/ml *Aspergillus* chitin (*n* = 9) or (B) with the different dosages 1, 10, and 100 µg/ml *Aspergillus* chitin in the presence of human serum (*n* = 3). (C) IL-1β and IL-1Ra transcription was measured by qPCR after 4 h of stimulation in the presence of human serum (*n* = 3). Statistical analysis was performed using a (A) Wilcoxon signed-rank test or (C) paired *t* test (*, *P* < 0.05; ***, *P* < 0.001; ns, not significant).

The chitin samples purified from *A. fumigatus* cell wall contained 70% of the particles showing sizes of <0.5 µm (Fig. 1) and a degree of acetylation of ~91% (see Fig. S1 in the supplemental material). Purity of the chitin was ensured as follows. First, the 3% of β-(1,3)-glucan still remaining bound to chitin after the final chemical extraction was removed by an endo-β-(1,3)-glucanase treatment (see Materials and Methods). Second, the absence of endotoxin contaminations was ensured by the pretreatment of chitin suspension with polymyxin B. In order to identify immunological function of chitin, human PBMCs were stimulated *in vitro* with chitin in the presence or absence of human serum. The proinflammatory cytokines IL-1β, tumor necrosis factor alpha (TNF-α), and IL-6 (Fig. 2A) were not detectable in the cell culture supernatant by enzyme-linked immunosorbent assay (ELISA) in the presence or absence of chitin. In addition, the anti-inflammatory cytokine IL-10 was not induced by chitin, neither on a protein level (Fig. 2A) nor on a transcription level, while lipopolysaccharide (LPS) and LPS with chitin induced high transcription of IL-10 (Fig. 2B). Although the level of IL-8 was high in the presence of chitin and serum, serum without chitin also induced a high level of IL-8, indicating that this high level of IL-8 is not due to the presence of chitin (Fig. 2C). However, chitin induced significant amounts of IL-1 receptor antagonist (IL-1Ra) compared to the medium control (Fig. 3A), in a dose-dependent manner (Fig. 3B). A kinetic analysis of IL-1Ra mRNA expression showed that IL-1Ra transcription peaked after 4 h of exposure of cells to chitin (Fig. 3C). The absence of transcription of IL-1β confirmed the lack of detection of this cytokine in the culture supernatant (Fig. 2A). In conclusion, serum was required for the induction of IL-1Ra transcription and release by PBMCs upon chitin stimulation.

### Dectin-1, TLR2, TLR4, MR, and NOD2 are not involved in chitin-induced IL-1Ra induction.

**FIG 4:**
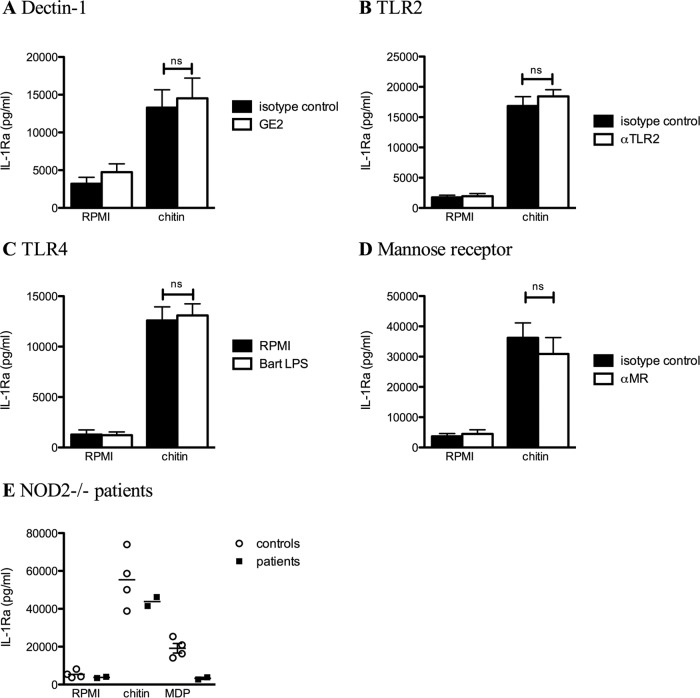
Dectin-1, TLR2, TLR4, MR, and NOD2 are not involved in the chitin-induced IL-1Ra. (A to E) Chitin-induced IL-1Ra in culture supernatants of PBMCs of healthy volunteers stimulated in the absence or presence of a neutralizing antibody or molecule like (A) GE2 for blocking dectin-1 (*n* = 5), (B) anti-TLR2 for blocking TLR2 (*n* = 6), (C) *Bartonella* (Bart) LPS for blocking TLR4 (*n* = 8), and (D) anti-MR for blocking the mannose receptor (*n* = 12), or (E) two NOD2-deficient patients. IL-1Ra was measured in the cell culture supernatant by ELISA. Statistical analysis was performed with the Wilcoxon signed-rank test. ns, not significant.

Subsequently, we aimed to identify the receptor that recognizes chitin that mediates IL-1Ra production by stimulation of PBMCs with chitin. Blocking dectin-1 with GE2 antibody did not affect chitin-induced IL-1Ra production (Fig. 4A). In addition, blocking TLR2 or TLR4 also did not influence IL-1Ra production by chitin (Fig. 4B and C). Since mannose receptor (MR) and NOD2 have been described as recognition receptors for *Candida albicans*-derived chitin (11), we stimulated PBMCs with chitin and human serum in the presence of an MR neutralizing antibody as well as stimulated PBMCs of NOD2-deficient patients with chitin and the NOD2 ligand *N*-acetylmuramyl-ananyl-d-isoglutamine (MDP). Blocking MR did not result in significant differences of the production of IL-1Ra (Fig. 4D), and as depicted in Fig. 4E, MDP stimulation did not result in any IL-1Ra production in the NOD-deficient patients, while chitin induced IL-1Ra in equal amounts as in the healthy control. In conclusion, chitin-induced IL-1Ra is dectin-1, TLR2 and -4, MR, and NOD2 independent.

### Immunoglobulin opsonization mediates chitin-induced IL-1Ra.

**FIG 5:**
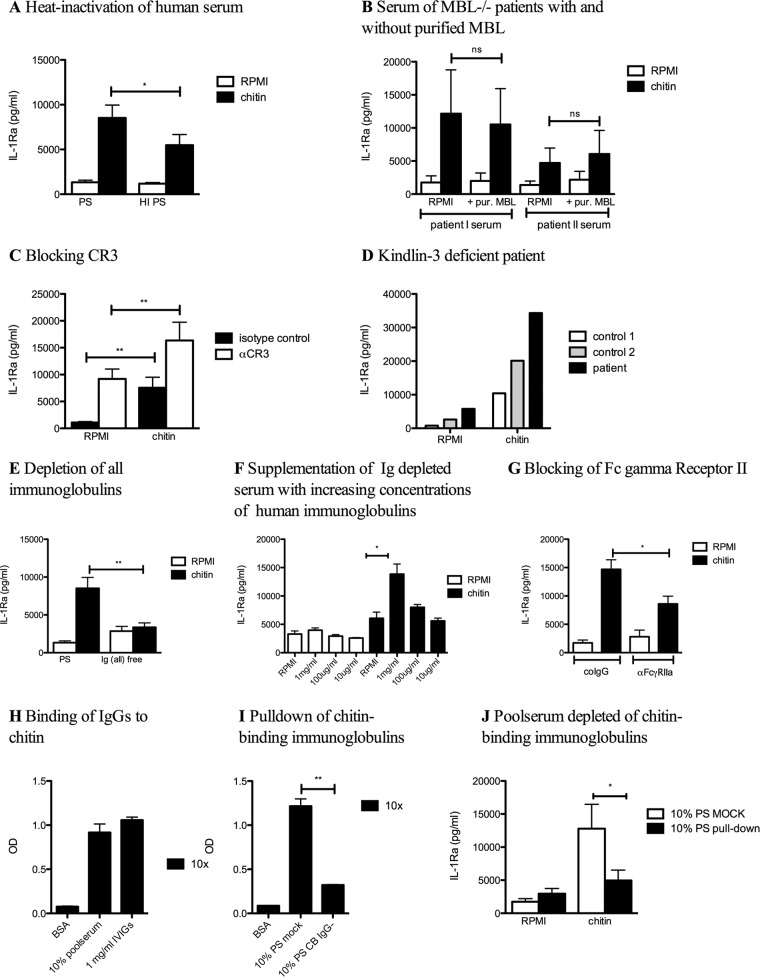
Complement and immunoglobulin opsonization mediate the chitin-induced IL-1Ra. PBMCs from healthy volunteers were stimulated with RPMI or chitin in the presence of (A) pooled serum (PS) or heat-inactivated pooled serum (HI PS) (*n* = 11), (B) MBL-deficient serum with and without additional purified MBL (*n* = 3), or (C) isotype control or CR3 neutralizing antibody (*n* = 8). (D) PBMCs from a kindlin-3-deficient patient or two controls were stimulated with chitin. (E) PBMCs from healthy volunteers were stimulated with RPMI or chitin in the presence of pooled serum or immunoglobulin-depleted serum (Ig^−^) (*n* = 11) or (F) Ig-depleted serum supplemented with decreasing concentrations of human immunoglobulins (IVIGs) (1 mg/ml, 100 µg/ml, and 10 µg/ml) (*n* = 6). (H and I) Binding of IVIGs to chitin was determined by coating an ELISA plate with chitin and a secondary incubation with (H) 10% pooled serum or 1 mg/ml IVIGs or (I) 10% pooled serum after mock treatment or after pulldown of chitin-binding immunoglobulins, and the OD was measured (*n* = 6). (G and J) PBMCs from healthy volunteers were stimulated with RPMI or with chitin in the presence of (G) an anti-Fc-γRII antibody or the isotype control (*n* = 3) or (J) in the presence of mock-treated pooled serum or with pooled serum depleted from chitin-binding immunoglobulins. (A to F, G, and J) IL-1Ra was measured in the cell culture supernatant by ELISA. Statistical analysis was performed with the Wilcoxon signed-rank test (*, *P* < 0.05; **, *P* < 0.01; ns, not significant).

Since none of the PRRs known to recognize fungi was identified as the receptor for the recognition of chitin to induce IL-1Ra, while human serum was crucial, we engaged in additional experiments to decipher the roles of different components present in human serum. In the presence of heat-inactivated serum, IL-1Ra was still induced, although there was a lower induction than with non-heat-inactivated serum (Fig. 5A). Since mannose-binding lectin (MBL) is an important polysaccharide-binding component, we stimulated PBMCs of healthy controls with chitin in the presence of MBL-deficient serum. However, IL-1Ra was still induced by sera exempt of MBL (Fig. 5B). CR3 is a known receptor recognizing complement-opsonized particles (12) and β-glucan (13, 14). IL-1Ra production was not abolished and even significantly increased when CR3 was blocked (Fig. 5C). To further validate this finding and avoid a masking effect of the blocking antibodies inducing IL-1Ra in combination with chitin, PBMCs isolated from a patient deficient for kindlin-3 (which is crucial for maximal CR3 signaling [15]) were stimulated with chitin. Comparable to CR3 blocking, kindlin-3 deficiency did not result in diminished IL-1Ra levels (Fig. 5D).

In contrast to the partial effects observed with heat inactivation of the serum, the induction of IL-1Ra by chitin was completely abolished when serum was depleted of all immunoglobulins (Fig. 5E). This result suggested that IgGs are responsible for the IL-1Ra induction by chitin. To demonstrate this, human immunoglobulins for intravenous (i.v.) administration (IVIGs) were added to the immunoglobulin-free serum, wherein IL-1Ra production could be restored in a dose-dependent manner, indicating that immunoglobulins are required for the induction of IL-1Ra by chitin (Fig. 5F). Fc-γ receptors recognize the Fc part of IgG and thus recognize IgG opsonized pathogens and particles. An Fc-γ receptor II (Fc-γRII) neutralizing antibody significantly reduced chitin-induced IL-1Ra production (Fig. 5G). To further elucidate whether chitin was directly bound and opsonized by IgG, we incubated 10% diluted serum or 1 mg/ml IVIGs in a polystyrene plate coated with chitin and subsequently detected bound IgG with an anti-Fc antibody. A significant amount of IgGs bound to chitin in both samples (Fig. 5H). To further elucidate whether the IL-1Ra induction was caused by immunoglobulins binding specifically to chitin, human serum was depleted from chitin-binding immunoglobulins. The efficacy of the pulldown experiment was tested by ELISA, resulting in a reduction of 73.5% of chitin-binding antibodies (Fig. 5I). Stimulation of PBMCs with chitin in the presence of serum depleted of chitin-binding IgGs resulted in a similar significantly lower IL-1Ra induction (Fig. 5J).

### Chitin-induced IL-1Ra is dependent on actin polymerization and signals via spleen tyrosine kinase and PI3K.

**FIG 6:**
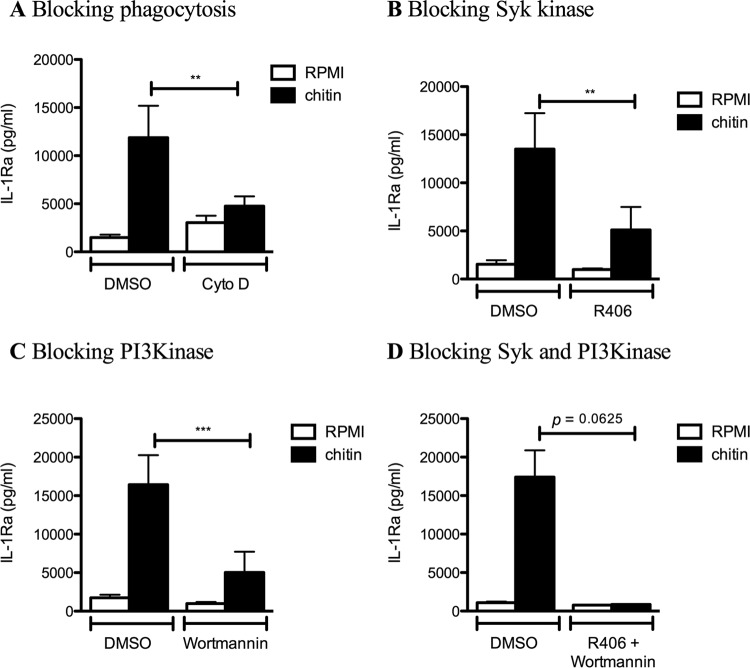
Chitin-induced IL-1Ra is dependent on actin polymerization and signals via Syk and PI3K. (A to D) PBMCs from healthy volunteers were stimulated with RPMI or chitin in the presence of (A) cytochalasin D actin depolymerization agent (*n* = 13), (B) the Syk kinase inhibitor R406 (*n* = 10), (C) wortmannin PI3K inhibitor (*n* = 11), or (D) the combination of R406 and wortmannin (*n* = 5), and IL-1Ra was measured in the cell culture supernatant by ELISA. Statistical analysis was performed with the Wilcoxon signed-rank test (**, *P* < 0.01; ***, *P* < 0.001).

Having identified opsonization with IgG as the main mechanism for the IL-1Ra induction by chitin, we further deciphered whether phagocytosis and the downstream signaling of Fc-γ receptor are needed for the induction of IL-1Ra by chitin. We stimulated PBMCs with chitin and human serum and blocked either the actin polymerization with cytochalasin D (Fig. 6A) or the downstream-signaling kinases of the Fc-γ receptor spleen tyrosine kinase (Syk) (Fig. 6B) or PI3K (Fig. 6C) or the combination of Syk and PI3K. All resulted in a significant reduction of IL-1Ra, while IL-1Ra was completely abolished when Syk and PI3K were blocked simultaneously (Fig. 6D). Taken together, these findings indicate that recognition and phagocytosis via the Fc-γ receptor with subsequent Syk kinase and PI3K activation mediates the IL-1Ra induction by chitin.

### Chitin synergizes with Syk-independent PRR pathways, leading to IL-1β production that is dependent on immunoglobulins, Syk, and PI3K.

**FIG 7:**
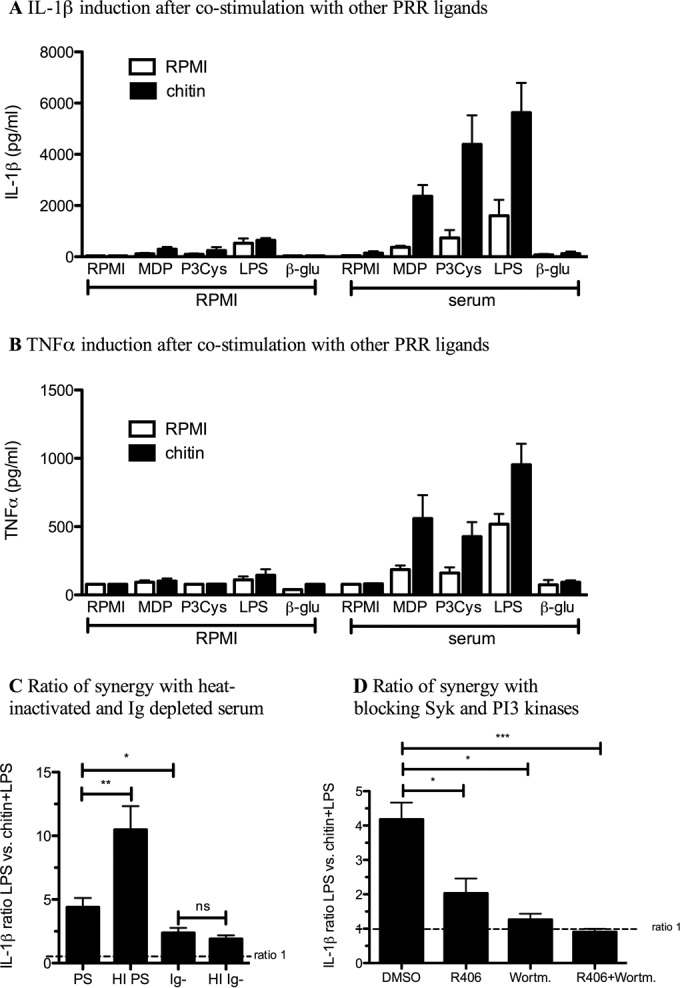
Chitin synergizes with PRR ligands not activating Syk, leading to high IL-1β production dependent on immunoglobulins, Syk, and PI3K. PBMCs from healthy volunteers were stimulated with (A and B) chitin, MDP, Pam3Cys, LPS, or β-glucan alone or with the combination of PRR ligands with chitin in the absence or presence of human pooled serum (*n* = 5 to 18) or (C and D) with LPS and LPS together with chitin, either (C) in the presence of heat-inactivated (HI) pooled serum, Ig-depleted serum (Ig^−^), or heat-inactivated Ig-depleted serum (HI Ig^−^) (*n* = 9) or (D) after blocking Syk kinase or PI3K alone or in combination (*n* = 5 to 7) (C and D). The ratio between LPS-induced IL-1β and chitin plus LPS-induced IL-1β was calculated. (A to D) IL-1β or TNF-α (B) was measured in the cell culture supernatant by ELISA. Statistical analysis was performed with the Wilcoxon signed-rank test (*, *P* < 0.05; **, *P* < 0.01; ***, *P* < 0.001; ns, not significant).

Since the immune system is probably never confronted in nature with the highly purified chitin alone, but rather in the setting of different PRR ligands of the *A. fumigatus* conidia and hyphal cell wall, we stimulated PBMCs with chitin in the absence and presence of serum in combination with the NOD2, TLR2, TLR4, and dectin-1 ligands. In the absence of serum, no cytokines were induced. All non-Syk-dependent PRR pathways stimulated with ligands, such as MDP (NOD2 pathway), Pam3Cys (TLR2 pathway), and LPS (TLR4 pathway) synergized with chitin to induce higher production of IL-1β and TNF-α, while stimulation with β-glucan (dectin-1/Syk pathway) combined with chitin did not induce IL-1β (Fig. 7A), TNF-α (Fig. 7B), or IL-6 (see Fig. S1 in the supplemental material). In contrast, chitin did not have synergistic effects on the LPS-induced IL-1Ra production; instead, LPS combined with chitin-induced IL-1Ra additively (see Fig. S3 in the supplemental material). Next, we wanted to elucidate whether similar pathways responsible for IL-1Ra induction by chitin were mediating the synergistic response of chitin with other PAMPs. Using heat-inactivated serum, we observed a significantly higher ratio of LPS versus LPS/chitin-synergistic induction of IL-1β, while this ratio was significantly lower in the presence of Ig-depleted serum. Further heat inactivation of the Ig-depleted serum did not change the ratio (Fig. 7C). In addition, blocking Syk and PI3K in PBMCs reduced the synergistic effect of chitin on LPS stimulation significantly, and the combination of both blockers completely inhibited the effect (Fig. 7D). Collectively, these data suggest synergism induced by chitin is also immunoglobulin/FcR dependent and provide evidence for cross talk between chitin-induced FcR signaling and other PRR pathways, with the exception of dectin-1.

## DISCUSSION

No pattern recognition receptor or signaling pathway activated by chitin has been identified in human immune cells to date, and immunostimulatory capacities range from absence of effects to anti-inflammatory and even proinflammatory properties (16). In the present study, we describe a mechanism by which chitin can induce immune responses in human immune cells and identify that chitin has both anti-inflammatory properties by inducing IL-1Ra and proinflammatory effects by inducing IL-1β/TNF in synergy with Syk-independent PRR pathways. Interestingly, none of the previously proposed chitin receptors, such as TLR2, MR, NOD2, or dectin-1, were involved in the induction of IL-1Ra in human PBMCs, but phagocytosis and recognition via the Fc-γRII with subsequent Syk and PI3K signaling of IgG-opsonized chitin was identified as the signaling pathway. Strikingly, the effect was only observed with chitin particles in the presence of immunoglobulins. Therefore, we propose that chitin has a dual immunological function by dampening and controlling immune responses together with immunoglobulins via the induction of IL-1Ra on one hand and orchestrating proinflammatory responses when chitin is presented in combination with PAMPs on the other.

Purified *Aspergillus* chitin does not induce any proinflammatory cytokines in PBMCs both in the absence and in the presence of human serum. This is in line with former studies by Mora-Montes et al. and Bueter et al. (16, 17), which described chitin as an immunologically inert particle, not inducing TNF-α, IL-1β, IL-6, or IL-10 production (17) or inflammasome activation (18). This was observed in several cell types, such as murine bone marrow-derived macrophages, M1 and M2 macrophages, as well as mouse peritoneal cells, mouse bone marrow-derived dendritic cells (DCs), and human PBMCs (18). Recently, it has been reported that chitin derived from different fungal species or crab shells induced IL-10 and TNF-α in human PBMCs in a dose-dependent manner (11). Several differences could account for this conflicting observation. The chitin particles in our study were mostly smaller than 1 µm, while the previously described IL-10-inducing chitin particles were between 1 and 10 µm (11). In addition, the use of *Aspergillus* to isolate chitin and the different chitin purification method could account for the differences observed in cytokine responses. The presence of a contaminant even at low dose in the chitin sample can have a significant effect on the response to chitin since we have shown that polysaccharides can have an opposite effect from chitin on the immune response when tested in combination with chitin.

While former studies focused on IL-10 as a potent anti-inflammatory cytokine, less attention has been paid to another potent endogenous anti-inflammatory cytokine, namely, IL-1Ra. In this study, stimulation of PBMCs with chitin in the presence of human serum resulted in IL-1Ra transcription and production. The fine-tuned balance of pro- versus anti-inflammatory cytokines of the IL-1 family is important during systemic inflammation, and several reports point to a crucial role for a balanced IL-1 response to prevent *Aspergillus*-induced pathology (19–21).

Since the MR has previously been reported to be involved in chitin-induced immune responses in mice (11), we investigated mannose-binding lectin (MBL), which shares homology with the MR, as well as blocking the MR on PBMCs. However, MBL and MR were not involved in IL-1Ra induction by chitin. Although CR3 plays a crucial role in mediating adaptive T cell responses against *Aspergillus* conidia (22, 23), this receptor was also not involved in chitin-induced IL-1Ra. Moreover, stimulation of PBMCs of a kindlin-3-deficient patient (defective CR3 signaling [15]) with chitin revealed an intact IL-1Ra pathway. These data suggest that CR3 and MBL are not involved in chitin-induced IL-1Ra production. However, the fact that IL-1Ra induction by chitin was partly dependent on heat inactivation suggests a role for the complement system in the induction of IL-1Ra by chitin. Since Agarwal et al. had shown in an earlier study that chitin is a poor inducer of the alternative complement pathway (24), the role of complement activation seen in this study could be caused by activation of the classical pathway. Elucidation of this should be part of future studies.

In contrast to complement, immunoglobulins were essential for mediating the induction of IL-1Ra induced by chitin. Chitin showed direct interaction with immunoglobulins, and the replenishment of IVIGs in Ig-depleted serum restored IL-1Ra induction. In addition, IL-1Ra was significantly lower when serum was depleted from chitin-binding immunoglobulins, suggesting that the presence of anti-chitin-specific IgGs mediates the IL-1Ra induction. Fc-γ receptors can be activated by binding to immunoglobulin-opsonized particles and subsequent internalization and receptor clustering (25). Fc-γRII has low affinity for monomeric IgG and only recognizes IgG complexes (26, 27), which might be similar to the ones artificially created by the binding of many IgGs to long chitin molecules. Blocking Fc-γRII resulted in a significant decrease of the induction of IL-1Ra by chitin, suggesting that chitin opsonized with IgGs triggers Fc-γII receptor signaling, subsequently leading to IL-1Ra. Moreover, we identified that IL-1Ra induction of opsonized chitin was dependent on phagocytosis, Syk, and PI3K activation.

Other polysaccharides present in the cell wall of *Aspergillus*, such as galactosaminogalactan and β-glucan, can also induce IL-1Ra (19, 28). However, both polysaccharides do not require human serum for the induction of IL-1Ra. Interestingly the induction of IL-1Ra by β-glucan was shown to be independent of dectin-1 but dependent on PI3K (29). Therefore, it might be that the PI3K-Akt pathway is the final common pathway essential for IL-1Ra induction by polysaccharides, but that this pathway can be induced via different mechanisms, depending on the ligand. Furthermore, it has been shown that insoluble fibrillar polysaccharides can induce potent inflammatory responses by engaging multimerization of pattern recognition receptors (PRRs) and formation of supramolecular PRR complexes, which is known as the fibril hypothesis (30). We observed striking effects when cells were stimulated with opsonized chitin in the presence of other PRR ligands. Ligands for TLR2, TLR4, and NOD2 mediated potent synergistic effects on proinflammatory cytokines such as IL-1β. Interestingly, the combination of chitin with β-glucan, the ligand for dectin-1, did not induce IL-1β production, not even in the presence of human serum. Recently, cross talk between Fc-γ receptors and other TLRs has been described (26). Vogelpoel et al. observed synergism with TLR2 and TLR4 ligands, but not with dectin-1 ligands, which is in line with the data from our study. Whether this is because Fc-γ receptors and dectin-1 both signal via Syk-PI3K-Akt needs to be further elucidated (31, 32). Since the synergism was dependent on IgGs and PI3K signaling, it is tempting to speculate that independent of a specific receptor, opsonized polysaccharides can induce potent synergistic proinflammatory responses via Fc-γ receptor activation when other proinflammatory ligands are present.

Studying the role of chitin-specific antibodies during aspergillosis might lead to new insights that could be relevant for the clinical setting. Indeed, an increase in antichitin antibodies has been shown in immunocompetent patients with chronic pulmonary aspergillosis and allergic aspergillosis (S. Raj and J.-P. Latgé, unpublished data). The role of the level of antibodies on the immune response toward chitin should be investigated in the future.

Another important question is how the opsonization of chitin with specific antibodies could influence disease severity. Only few data exist; however, Kin et al. have shown that antibodies against bacterial polysaccharides can bind to chitin and dampen the overall immune response to chitin (33). In addition, since we provide evidence that chitin induces a different immune response when tested alone or with other PAMPs and since we know that the different polysaccharides of *A. fumigatus* will activate differently inflammatory responses, it is now of interest to test the effect of combinations of the cell wall polysaccharides to see if these combination are neutral, antagonistic, or synergistic.

**FIG 8:**
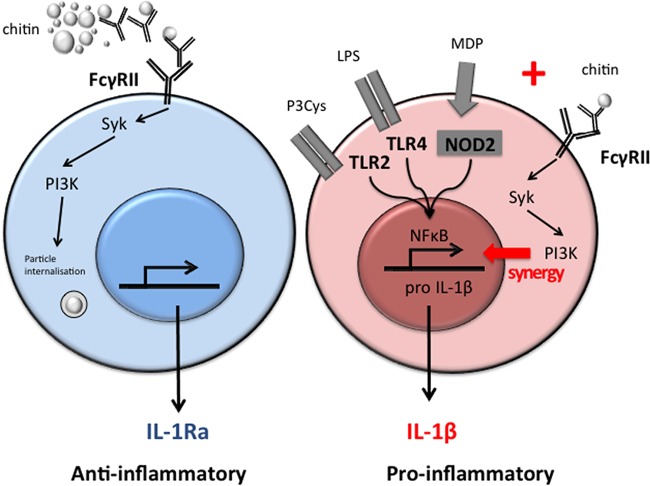
Model of chitin-induced anti-inflammatory IL-1Ra and proinflammatory IL-1β responses. IgG-opsonized chitin is recognized by the Fc-γ receptor and uptake induced via the Syk/PI3K pathway, which results in isolated induction of IL-1Ra. In the presence of other Syk-independent PRR ligands, like Pam3Cys (P3Cys), LPS, and MDP, IgG-opsonized chitin induces IL-1β in a synergistic manner.

In conclusion, we have identified a novel mechanism through which chitin has strong anti-inflammatory effects and in the setting of other PAMPs can boost proinflammatory responses (Fig. 8). These responses were critically dependent on immunoglobulins, Fc-γ receptor signaling, and phagocytosis. These data might explain why the responses reported by chitin are so diverse. Moreover, since immunoglobulins might also influence other polysaccharide-induced immune responses, future studies need to focus on immunoglobulins and FcR signaling when investigating fungal or fungal-cell wall component-induced immune responses.

## MATERIALS AND METHODS

### Volunteers and patients.

Blood was collected from healthy volunteers or patients by venous blood puncture. Two NOD2-deficient individuals had the homozygous NOD2 insertion of a cysteine at position 1007 (rs2066847) that results in a frameshift of the coding sequence, which was analyzed as described previously (34). Serum was used from two patients who had a deficiency in mannose-binding lectin (MBL) with serum MBL levels below 0.04 mg/liter. One patient had the autosomal recessive mutation in *FERMT3*, the gene encoding kindlin-3, leading to the leukocyte adhesion deficiency type III (35, 36).

### Ethics statement.

All experiments were performed and conducted in accordance with good clinical practices, the Declaration of Helsinki, and the approval of the Arnhem-Nijmegen Ethical Committee (no. 2010/104). Blood from volunteers and patients was taken after written informed consent was obtained.

### PRR ligands, blockers, and other stimuli.

*E. coli* lipopolysaccharide (LPS [1 ng/ml]) (TLR4 ligand [*E. coli* serotype O55:B5]) was obtained from Sigma-Aldrich, St. Louis, MO), Pam3Cys (1 µg/ml) (TLR2 ligand) was obtained from EMC Microcollections, Tübingen, Germany, and *N*-acetylmuramyl-ananyl-d-isoglutamine (MDP [5 μg/ml]) (NOD2 ligand) was obtained from Sigma-Aldrich. Fungal cell wall β-(1,3)-glucan (10 µg/ml) (dectin-1 ligand) was kindly provided by David L. Williams (East Tennessee State University, Johnson City, TN). This polysaccharide was isolated from *C. albicans* yeast cells and was verified to be exempt of proteins. The suspension contained water-insoluble microparticles (1 to 5 µm) (37).

The dectin-1 inhibitor GE2 was a kind gift from Gordon Brown (University of Aberdeen, Scotland). *Bartonella quintana* LPS was prepared and purified as described elsewhere (38) and used as a TLR4 inhibitor (100 ng/ml) (22). Isotype control mouse IgG1 (10 µg/ml) and anti-TLR2 (10 µg/ml) were obtained from eBioscience, Halle-Zoersel, Belgium. Isotype control goat IgG (10 µg/ml), anti-human β_2_-integrin (anti-CR3 [10 µg/ml]), and isotype control mouse IgG2b (10 µg/ml) were obtained from R&D Systems Minneapolis, MN. Isotype control mouse IgG1κ (10 µg/ml) and anti-human mannose receptor (MR) (anti-human CD206 [10 µg/ml]) were obtained from BioLegend, San Diego, CA. Anti-human CD32 (anti-Fc-γ receptor II [10 µg/ml]) was obtained from StemCell Technologies SARL, Grenoble, France. Wortmannin (100 ng/ml, dissolved in dimethyl sulfoxide [DMSO]) was obtained from Sigma. R406 Syk-kinase inhibitor (5 µM, dissolved in DMSO) was obtained from InvivoGen, Toulouse, France. Cytochalasin D (10 µg/ml, dissolved in DMSO) and polymyxin B (2 µg/ml, preincubated for 2 h with chitin at 37°C) were obtained from Sigma-Aldrich. Human immunoglobulins for intravenous administration (IVIG) were obtained from Nanogam, Sanquin, Amsterdam, The Netherlands, and were dialyzed in phosphate-buffered saline (PBS) before use. Mannose-binding lectin (MBL) was purified from human plasma, as described in a previous study (39).

### Purification and characterization of chitin from the *A. fumigatus* cell wall.

Chitin was isolated from the *A. fumigatus* cell wall (*A. fumigatus* CEA17_Δ*akuB*^KU80^ strain [40], with mycelia collected after 20 h of growth at 37°C in liquid Sabouraud medium) according to the method described earlier (17, 41). However, the resultant chitin showed ~3% β-(1,3)-glucan contamination when checked by gas chromatographic analyses (42). This contamination, which could have important immunological consequences, was cleared upon recombinant endo-β-(1,3)-glucanase treatment. In brief, chitin preparation (5 mg in 0.5 ml of 50 mM acetate buffer, pH 6.0) was treated with endo-β-(1,3)-glucanase (20 µl containing 5 µg protein) at 37°C overnight followed by centrifugation and checking the supernatant for reducing sugar released by *p*-aminobenzoic acid assay using 4-hydroxy-benzhydrazide (43). This was repeated until no more reducing sugar was released into the supernatant. Furthermore, the chitin preparation was washed thoroughly with sterile water, and the absence of β-(1,3)-glucan was confirmed by gas chromatographic analysis. The degree of acetylation in the chitin preparation was determined by three different methods. (i) The first method was the Cibacron brilliant red 3B-A dye binding method (44). (ii) The second method was a UV spectrophotometry method using dual standards (glucosamine [GlcN] hydrochloride and *N*-acetyl-glucosamine [GlcNAc]). For UV analysis, the chitin was solubilized upon ultrasonication. A stock aqueous chitin suspension (1 mg/ml) was prepared by ultrasonication using a Soniprep 150 probe sonicator (MSE, London, United Kingdom). The sonicator was set to 50% power and tuned to 50%, reaching an amplitude of 16 μm for 30 s. The sonication reduced the molecular weight while not affecting the degree of acetylation (45, 46). (iii) The third method was a Fourier transform infrared spectroscopy (FT-IR) method performed using the absorption ratio *A*_1655_/*A*_3450_ (47, 48). For FT-IR, dry chitin was suspended uniformly between IR windows, and the spectrum (1,400 to 4,000 cm^− 1^) was recorded in a Jasco FT/IR-6100 apparatus, subtracting the spectra of air.

Experiments were performed with four different batches of chitin purified from the *A. fumigatus* mycelial cell wall, with a final concentration of 10 µg/ml.

To determine the size of the chitin particles, the chitin suspension was subjected to flow cytometry (FC500 flow cytometer [Beckman Coulter]) and compared with reference beads of 0.1 and 0.25 µm from Thermo Scientific or 3, 6, and 10 µm (Flow Check Pro Fluorospheres) from Beckman Coulter. The data were analyzed using Kaluza Analysis version 1.3 (Beckman Coulter).

### PBMC isolation.

Venous blood was drawn in 10 ml EDTA tubes. The blood was diluted 1:1 with phosphate-buffered saline (PBS). Subsequently PBMCs were isolated using Ficoll-Paque (GE Healthcare, Zeist, The Netherlands) density gradient centrifugation. The PBMC layer was collected and washed twice in cold PBS. Cells were reconstituted in RPMI+, consisting of RPMI 1640 culture medium (Dutch modification [Gibco, Invitrogen, Breda, The Netherlands]) supplemented with 10 µg/ml gentamicin, 10 mM l-glutamine, and 10 mM pyruvate (Gibco). The cells were counted with a particle counter (Beckmann Coulter, Woerden, The Netherlands), and the concentration was adjusted to 1 × 10^7^ cells/ml.

### PBMC stimulation.

PBMCs were plated in a 96-well plate (Corning, NY) at a final concentration of 2.5 × 10^6^/ml in an end volume of 200 µl per well. Stimulations were performed in the presence of 10% human serum, either not depleted or depleted of all immunoglobulins (BBI Solutions, Cardiff, United Kingdom). Serum was either complement active, if not otherwise indicated, or heat inactivated by incubation for 30 min at 56°C in a water bath according to a commonly used protocol (49). After 1 h of preincubation with inhibitor or medium, stimuli or medium was added. Cells were incubated at 37°C with 5% CO_2_, and after 24 h, supernatants were collected and stored at −20°C.

### Cytokine measurements.

IL-1β, tumor necrosis factor (TNF-α), IL-6, IL-8, IL-10, and IL-1Ra were measured in the cell culture supernatants using commercial ELISA kits (IL-1β, TNF-α, and IL-1Ra from R&D Systems, and IL-6, IL-8, and IL-10 from Sanquin) according to the instructions supplied by the manufacturer.

### Pulldown assay of chitin-binding immunoglobulins in human serum.

To deplete serum from chitin-binding immunoglobulins, 1 ml of RPMI supplemented with 10% serum containing either 1 mg chitin from a stock concentration of 10 mg/ml or an equal amount of distilled water (for mock treatment) was incubated overnight at 37°C and shaken at 130 rpm. On the next day, the chitin-containing or mock-treated suspension was centrifuged (20 min, 14,000 rpm, room temperature), and the supernatant without beads was recovered. The depletion efficacy was checked by a chitin-binding IgG ELISA.

### Chitin-binding IgG ELISA.

A polystyrene 96-well plate was coated with 10 µg/ml chitin (100 µl per well) overnight at room temperature. After being washed and blocked with PBS with 1% bovine serum albumin (BSA) for 1 h, wells were incubated with 10% serum in RPMI+, mock-treated serum, or depleted serum for 1 h. IgGs bound to chitin were detected by using an anti-human IgG (whole particle) labeled with peroxidase in a concentration of 1:1,000 (Sigma-Aldrich). The enzymatic reaction was started by adding the TMB (3,3′,5,5′-tetramethylbenzidine)- and H_2_O_2_-containing substrate, which was stopped after 10 min by 10% sulfuric acid solution. The optic density was measured by a photometric ELISA reader.

### qPCR.

RNA was isolated from 1 × 10^6^ PBMCs after stimulation for 4 h and 24 h with *Aspergillus* chitin in the presence of either medium or 10% human serum using Trizol reagent (Invitrogen) according to a protocol supplied by the manufacturer. RNA (500 ng) was reverse transcribed into cDNA using the iScript cDNA synthesis kit (Hercules, Bio-Rad Laboratories, CA). Quantitative PCR (qPCR) analysis was performed using SYBR green master mix (Applied Biosystems, Carlsbad, CA) and the Applied Biosystems 7300 real-time PCR system. As in the PCR protocol, the following conditions were used: 2 min at 50°C and 10 min at 95°C, followed by 40 cycles at 95°C for 15 s and 60°C for 1 min. For the amplification of human IL-1Ra (hIL-1Ra), the primers 5′ GCCTCCGCAGTCACCTAAT 3′ and 5′ TCCCAGATTCTGAAGGCTTG 3′ were used, and for the amplification of hIL-1β, the primers 5′ GCAACTGTTCCTGAACTCAACT 3′ and 5′ ATCTTTTGGGGTCCGTCAACT 3′ were used. To correct for differences in loading concentrations of RNA between the different conditions, qPCR results were corrected with the β2 microglobulin (β2m) housekeeping gene amplified using the primers 5′ ATGAGTATGCCTGCCGTGTG 3′ and 5′ CCAAATGCGGCATCTTCAAAC 3′. Primer efficacy was evaluated using a standard curve. IL-1Ra threshold cycle (*C_T_*) values were compared with the β2m *C_T_* by calculating the Δ*C_T_*, and the fold change was calculated relative to the RPMI-stimulated level to determine the effect of stimulation with chitin on IL-1Ra expression.

### Statistical analysis.

The Wilcoxon signed-rank test was used to determine differences between stimulation with and without inhibitors or between different sera. A *P* value of <0.05 was considered statistically significant as shown by asterisks in the figures (*, *P* < 0.05; **, *P* < 0.01; ***, *P* < 0.001). Graphs represent cumulative results from all performed experiments and are presented as means ± standard errors of the means (SEM). Data were analyzed with GraphPad Prism v 5.0.

## SUPPLEMENTAL MATERIAL

Figure S1  Measurement of the degree of acetylation of chitin. With IR, UV spectrometric, and dye binding measurements, different batches of chitin showed degrees of acetylation ranging between 89 and 94%. An average value of 91% from four different batches of chitin is presented. Download Figure S1, TIF file, 0.4 MB

Figure S2  IL-6 induction after costimulation with other PRR ligands. PBMCs from healthy volunteers were stimulated with chitin, MDP, and Pam3Cys alone and with the combination of PRR ligands with chitin in the absence or presence of human pooled serum (*n* = 5 to 18). Download Figure S2, TIF file, 0.6 MB

Figure S3  No synergy of chitin and LPS for IL-1Ra. PBMCs of healthy volunteers were stimulated with chitin and LPS alone and with the combination of chitin and LPS in the presence of human pooled serum (*n* = 6). IL-1Ra was measured in the cell culture supernatant by ELISA. Statistical analysis was performed with the Wilcoxon signed-rank test. Download Figure S3, TIF file, 0.9 MB
