# Supplementary figures and images for: Aspergillus Cell Wall Chitin Induces Anti- and Proinflammatory Cytokines in Human PBMCs via the Fc-γ Receptor/Syk/PI3K Pathway
Source: mBio. 2016 May 31;7(3):e01823-15. doi: 10.1128/mBio.01823-15 (PMC4895119; doi:10.1128/mBio.01823-15)

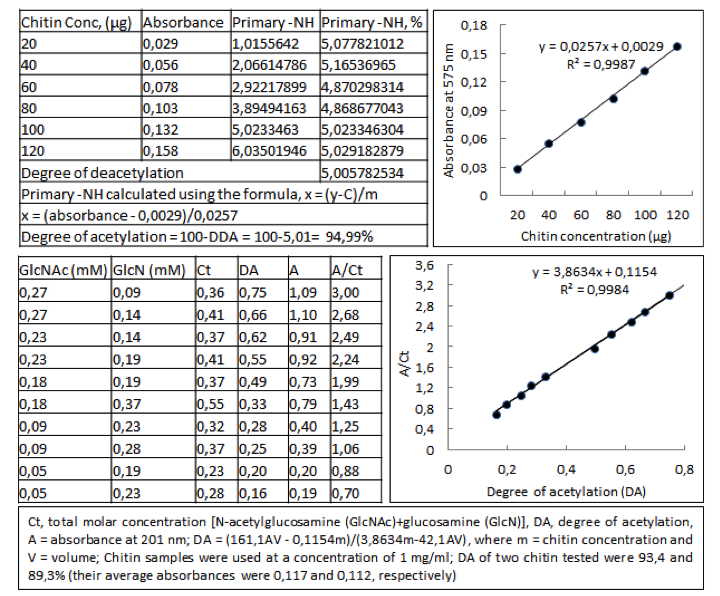

Supplement: Figure S1 — Measurement of the degree of acetylation of chitin. With IR, UV spectrometric, and dye binding measurements, different batches of chitin showed degrees of acetylation ranging between 89 and 94%. An average value of 91% from four different batches of chitin is presented. Download [file mbo003162839sf1.tif]

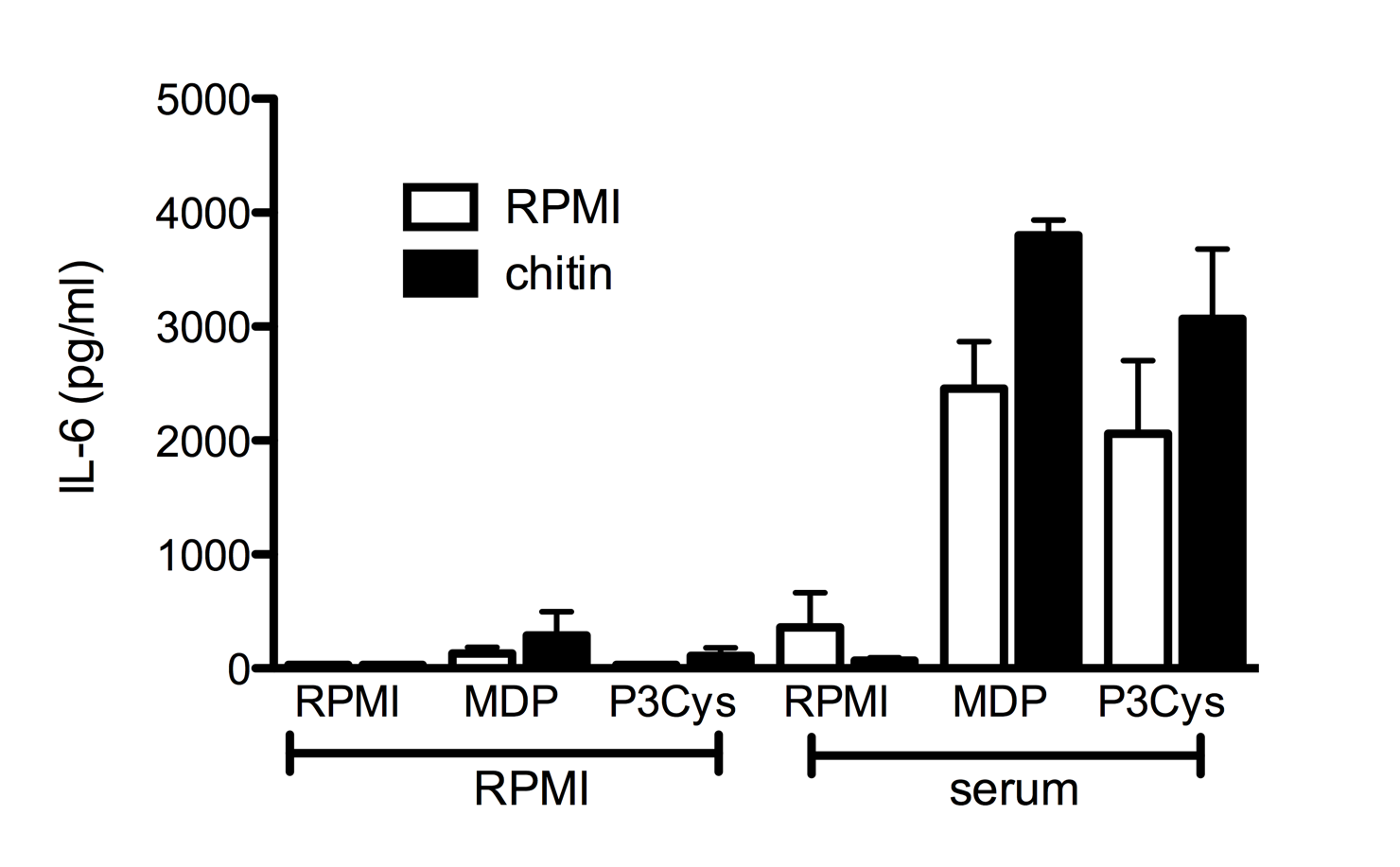

Supplement: Figure S2 — IL-6 induction after costimulation with other PRR ligands. PBMCs from healthy volunteers were stimulated with chitin, MDP, and Pam3Cys alone and with the combination of PRR ligands with chitin in the absence or presence of human pooled serum (n = 5 to 18). Download [file mbo003162839sf2.tif]

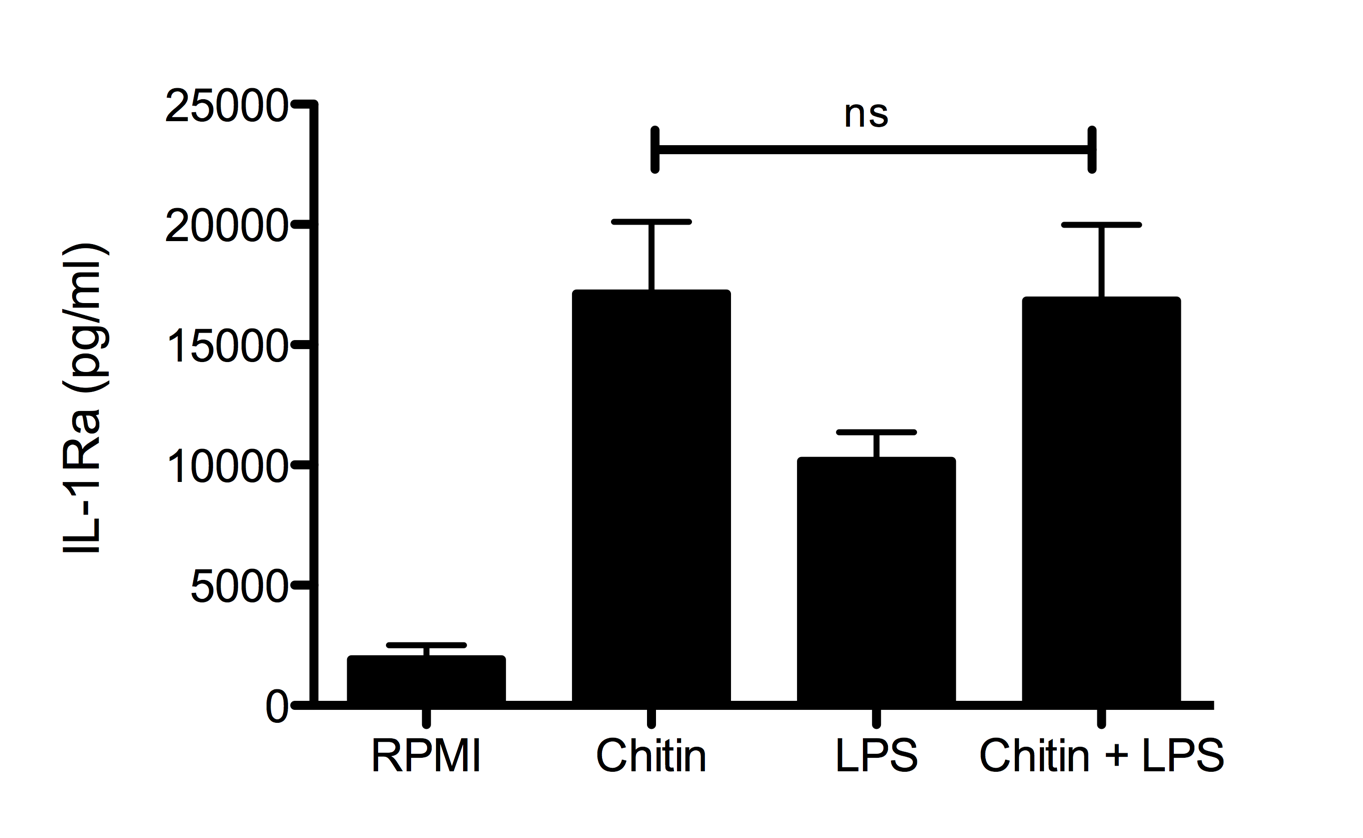

Supplement: Figure S3 — No synergy of chitin and LPS for IL-1Ra. PBMCs of healthy volunteers were stimulated with chitin and LPS alone and with the combination of chitin and LPS in the presence of human pooled serum (n = 6). IL-1Ra was measured in the cell culture supernatant by ELISA. Statistical analysis was performed with the Wilcoxon signed-rank test. Download [file mbo003162839sf3.tif]
